# Human Erythrocytes Exposed to Phthalates and Their Metabolites Alter Antioxidant Enzyme Activity and Hemoglobin Oxidation

**DOI:** 10.3390/ijms21124480

**Published:** 2020-06-24

**Authors:** Paulina Sicińska, Kinga Kik, Bożena Bukowska

**Affiliations:** Department of Biophysics of Environmental Pollution, Faculty of Biology and Environmental Protection, University of Lodz, Pomorska Str. 141/143, 90-236 Łódź, Poland; kinga.malinowska@unilodz.eu (K.K.); bozena.bukowska@biol.uni.lodz.pl (B.B.)

**Keywords:** phthalates, methemoglobin, reactive oxygen species, hydroxyl radical, superoxide dismutase, catalase, glutathione peroxidase

## Abstract

Phthalates used as plasticizers have become a part of human life because of their important role in various industries. Human exposure to these compounds is unavoidable, and therefore their mechanisms of toxicity should be investigated. Due to their structure and function, human erythrocytes are increasingly used as a cell model for testing the in vitro toxicity of various xenobiotics. Therefore, the purpose of our study was to assess the effect of selected phthalates on methemoglobin (metHb), reactive oxygen species (ROS) including hydroxyl radical levels, as well as the activity of antioxidative enzymes, such as superoxide dismutase (SOD), catalase (CAT), and glutathione peroxidase (GSH-Px), in human erythrocytes. Erythrocytes were incubated with di-n-butyl phthalate (DBP), butylbenzyl phthalate (BBP), and their metabolites, i.e., mono-n-butyl phthalate (MBP) and monobenzyl phthalate (MBzP), at concentrations ranging from 0.5 to 100 µg/mL for 6 or 24 h. This study shows that the analyzed phthalates disturbed the redox balance in human erythrocytes. DBP and BBP, at much lower concentrations than their metabolites, caused a statistically significant increase of metHb and ROS, including hydroxyl radical levels, and changed the activity of antioxidant enzymes. The studied phthalates disturbed the redox balance in human erythrocytes, which may contribute to the accelerated removal of these cells from the circulation.

## 1. Introduction

Nowadays, the list of substances that can have a serious impact on human health and the environment is systematically growing. The European Chemicals Agency (ECHA) has identified these harmful chemicals as substances of very high concern (SVHC). This list also includes phthalates (PAEs), which are the most commonly used plasticizers in the world. Phthalates have been qualified as endocrine-disrupting chemicals (EDCs), and several of them, including di-n-butyl phthalate (DBP) and butylbenzyl phthalate (BBP), have been inscribed in the Candidate List of SVHCs [1].

**Figure 1:**
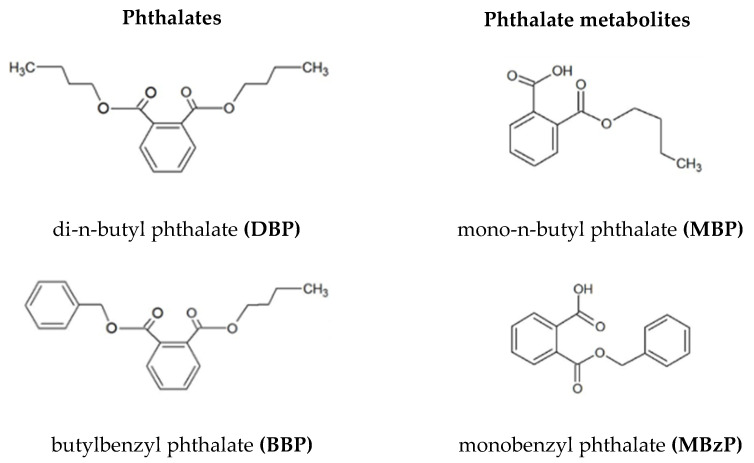
Chemical structures of the studied phthalates.

Due to their properties, DBP and BBP are widely used in the production of plastics and cosmetics and in the pharmaceutical industry [2,3,4]. Phthalates do not form covalent bonds with the substances they are being added to and thus they may migrate easily and enter food, water, air, cosmetics, and various products of everyday use [5,6,7]. These compounds enter the human body mainly via the enteral pathway (food, water, drugs) at about 7–10 µg/kg of body weight (BW)/day but also by inhalation (concentration in the air: BBP 0.058–3.97mg/m^3^, DBP 1.5–270 ng/m^3^) or through dermal contact with cosmetics (DBP max 0.594 ppm and BBP max 186.770 ppm) [7,8,9,10]. After entering the organism, DBP and BBP are decomposed by lipases and esterases to monoesters, such as mono-n-butyl phthalate (MBP) and mono-benzyl phthalate (MBzP) (Figure 1) [11,12].

Phthalates and their metabolites have been detected in humans around the world. In peripheral blood and umbilical cord blood, DBP has been detected in the concentration ranges from 0.051 to 7.67 μg/mL and from 0.0197 to 5.71 μg/mL, respectively [13,14]. In blood serum, DBP was found at a concentration of 0.77–12.50 ng/mL, and BBP at a concentration of 0.82–1.97 ng/mL [15]. DBP (0.25–269 μg/L) and MBzP (7.4–9.5 ng/mL) were determined in the urine of adults and children [16,17,18,19,20,21,22]. DBP and BBP were detected at approx. 2 μg/mL and 4 μg/mL, respectively, in hair samples [23].

Many epidemiological studies have shown that phthalates, after entering the body, cause disorders of the endocrine system. They can also contribute to the development of allergies, asthma, obesity, metabolic syndrome, type 2 diabetes, cardiovascular diseases, and malignant neoplasms, and all these disorders are associated with oxidative stress induction [24,25,26,27].

Oxidative stress is a disturbance of the balance between the formation and the removal of free radicals. It arises as a result of increasing reactive oxygen species (ROS) level, reduced amount of scavengers, and depletion of the activities of antioxidant enzymes [28]. Currently, most studies on oxidative stress under the influence of SVHCs, such as DBP, BBP, and their metabolites, are being conducted on plants or animals. In plants, these phthalates have been shown to disturb the activity of superoxide dismutase, catalase, and glutathione peroxidase, increase ROS level, and induce lipid peroxidation [29,30]. Similar changes of the same parameters have been observed in animals (earthworms, *Danio rerio*, mice, rats). These examples may indicate that these substances produce oxidative stress in various organisms [31,32,33,34,35,36].

There are few studies on the effect of phthalates, like DBP, BBP, MBP, and MBzP, on human blood cells. Literature data have shown that these phthalates cause hemolysis and eryptosis of human red blood cells (RBCs), may interact with hemoglobin [37,38], and induce apoptosis in human peripheral blood mononuclear cells (PBMCs) [39]. DBP and its metabolite MBP also exhibit cytotoxic potential and cause cytokine secretion in human PBMCs [40].

The aim of our study was to compare the effects of phthalates (DBP, BBP) and their metabolites (MBP, MBzP) on selected markers of oxidative stress (methemoglobin (metHb) and ROS, including hydroxyl radical (^•^OH) levels and the activity of antioxidative enzymes, including catalase (CAT), superoxide dismutase (SOD glutathione peroxidase(GSH-Px)) in human erythrocytes [41,42]. The tested compounds were mainly used at the concentrations from 0.5 μg to 10 μg/mL because phthalates have been found in human blood in concentrations from 0.0197 to 7.67 μg/mL [13,14]. In order to thoroughly determine the mechanism of action of phthalates, we also conducted studies at slightly higher phthalates concentrations, i.e., in the range of their pre-hemolytic concentrations, which are 20 μg/mL in the case of the parent compounds (DBP, BBP) and 100 μg/mL in the case of their metabolites (MBP, MBzP) [37]. Therefore, in this study, human RBC were finally treated with parent phthalates at concentrations ranging from 0.5 to 20 μg/mL and with their metabolites at concentrations from 0.5 to 100 μg/mL. Erythrocytes, in addition to their primary function of transporting oxygen from the lungs to tissues and carbon dioxide from tissues to the lungs, are involved in the transport and/or storage of toxic substances [43]. Hemoglobin (Hb), the main protein present in erythrocytes, can be oxidized directly by xenobiotics or indirectly by reactive oxygen species produced by xenobiotics transported by these cells [43,44]. Interactions of xenobiotics with Hb may lead to changes in Hb structure and loss of its function [45]. In addition, these cells have a very well developed antioxidant system [46]. Therefore, the conduction of vitro tests using human RBC is justified and may become an essential tool for the assessment of the toxicity of DBP, BBP, and their metabolites (MBP, MBzP) in the human organism [41,47].

## 2. Results

### 2.1. Hemoglobin Oxidation

After 24 h of incubation of the erythrocytes with increasing phthalates concentrations, an increase in metHb level was observed. DPB caused a statistically significant increase in the parameter studied starting from the concentration of 2.5 µg/mL, while BBP produced this effect from the concentration of 5 µg/mL (Table 1). A statistically significant increase in metHB level was observed in RBCs treated with MBP and MBzP only starting from a concentration of 50 µg/mL (Table 1).

### 2.2. ROS Levels

**Figure 2:**
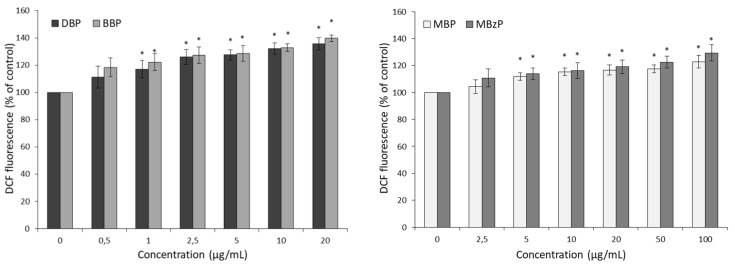
Changes in reactive oxygen species levels in control erythrocytes and in erythrocytes incubated for 6 h with DBP, BBP, MBP, and MBzP used at concentrations ranging from 0.5 to 100 μg/mL. (*) *p* < 0.05.

After 6 h of incubation of the erythrocytes with phthalates, an increase in ROS level was observed as compared to the control (100%). DBP and BBP caused a statistically significant increase in ROS levels starting from a concentration of 1 µg/mL and corresponding to 11.8% and 13.7%, respectively (Figure 2). For their metabolites, MBP and MBzP, a significant increase in ROS level starting from a concentration of 5 µg/mL and corresponding to 17.1% and 22.2%, respectively, was noted (Figure 2).

**Figure 3:**
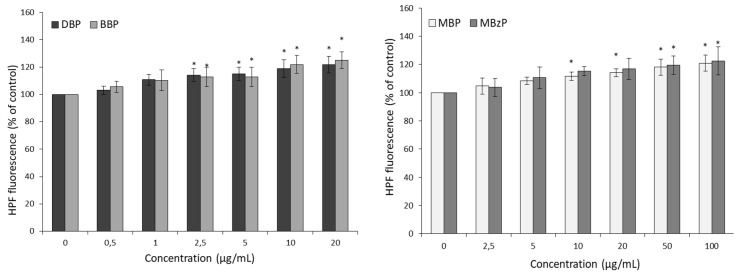
Changes in hydroxyl radical levels in control erythrocytes and in erythrocytes incubated for 6 h with DBP, BBP, MBP, and MBzP used at concentrations ranging from 0.5 to 100 μg/mL. (*) *p* < 0.05.

A statistically significant increase in 3′-(p-hydroxyphenyl)-fluorescein (HPF) oxidation (mainly ^•^OH formation) with respect to the control (100%) was observed in RBCs incubated with DBP and BBP at 2.5 μg/mL, corresponding to 11.7% and 15.2%, respectively. MBP starting from 10 μg/mL and MBzP starting from 50 μg/mL caused an increase in HPF fluorescence by 14.2% and 21.9%, respectively (Figure 3).

### 2.3. Superoxide Dismutase Activity

A statistically significant decrease in SOD activity after 24 h of incubation of the erythrocytes with DBP (74.8%) and BBP (66.2%) starting from a concentration of 2.5 μg/mL was observed. A similar decrease was observed for MBP and MBzP but starting from a higher concentration corresponding to 10 μg/mL (by 60.9% and 63.6%, respectively) (Table 2).

### 2.4. Catalase Activity

DBP and BBP, starting from a concentration of 5 µg/mL, caused a statistically significant increase in CAT activity by 36% and 24%, respectively. The phthalate metabolites caused statistically significant changes only starting from a concentration of 50 µg/mL (Table 2).

### 2.5. Glutathione Peroxidase Activity

A statistically significant decrease in the activity of this enzyme after 24 h of incubation of RBCs with the parent phthalates (starting from a concentration of 5 µg/mL) was observed, which was approximately 30%. For MBP and MBzP (starting from a concentration of 50 µg/mL), a statistically significant decrease of GSH-Px activity was noted, which was estimated to be 32% and 23%, respectively (Table 2).

## 3. Discussion

Many xenobiotics are able to generate ROS, increase redox reactions, and reduce the activity of the antioxidant system [41,48,49], therefore contributing to the generation of oxidative stress in cells, which often accompanies numerous diseases [25,50]. For this reason, we have evaluated the effect of phthalates, i.e., DBP and BBP and their metabolites like MBP and MBzP, on selected parameters of oxidative stress in human erythrocytes.

Phthalates have relatively high logarithmic values of the octanol–water partition coefficient (K_ow_) and demonstrate a good solubility in lipids, easily penetrating the erythrocyte membrane and thus entering cells by in vivo transport and diffusion processes [51,52]. Hemoglobin is the main component of RBCs and their only nonmembrane protein [45,46]. Therefore, phthalates entering RBCs may easily interact with Hb, altering its structure and function [45,52]. The administration of DBP and BBP to rats showed a decreased in the level of RBCs, Hb, and hematocrit [44]. A similar correlation was observed by Zhu et al. examining the blood from a group of pregnant women. The authors showed that an increase in the concentration of phthalate metabolites in the urine (including MBP and MBzP) correlated with a decrease in the amount of Hb in the blood and an increase of anemia in the women studied [52]. In vitro studies on the effects of DBP, BBP, and their metabolites on human erythrocytes have shown that these phthalates decreased erythrocyte viability (hemolysis) and induced eryptosis [37]. Hemolysis leads to a release of Hb from erythrocytes. This phenomenon can contribute to metHb^3+^ formation, which is incapable of oxygen transport as it binds to oxygen very tightly and does not release it in tissues, even when the partial pressure of oxygen is low [38,53,54,55].

Our study showed an increase in metHb level along with increasing phthalates concentrations, and DBP showed the strongest methemoglobinogenic activity (Table 1). The increase in metHb level could be due to a significant decrease of the activity of metHb reductase in erythrocytes incubated with the studied phthalates. Hemoglobin oxidation may also be the result of damage to the RBC membrane or excessive ROS production or may be associated with the direct interaction of the analyzed compounds with hemoglobin [49]. The experiments with bovine hemoglobin (BHb) reported by Chi et al. demonstrated that DBP could interact with BHb to form BHb–PAE complexes through one binding site in the central cavity of BHb and the creation of hydrophobic forces. The binding of PAEs to BHb could change the secondary structure of BHb, which may affect function of this protein [45]. Other studies on human hemoglobin (hHb) showed that the aromatic ring present in PAE significantly increased the binding strength between hHb and PAE but reduced the depth of the binding position in the hydrophobic space of hHb center. PAEs with a higher number of carbon atoms (which means higher hydrophobicity) have been shown to move deeper into the hydrophobic space of the hHb center and bind this protein at different sites [38]. MetHb formation suggests that phthalates target Hb and its heme groups. Heme degradation usually leads to an increase in the level of free iron ions, which are active in redox reactions and can react with H_2_O_2_ to form highly reactive hydroxyl radicals [56,57,58,59,60]. Therefore, we determined the level of ROS, including that of hydroxyl radical.

Some studies have shown that phthalates in young men induced ROS production, contributing to oxidative stress in Sertoli and Leydig cells, resulting in the inhibition of the spermatogenesis process and in the reduction of spermatozoid count [61,62]. Our study showed that DBP and BBP at a concentration as low as 1 µg/mL caused an increase in ROS formation (Figure 2). A similar tendency was observed by Yin et al., in mouse embryonic stem cells exposed to DBP [34]. Phthalates metabolites also caused an increase in ROS level but starting from a concentration of 5 µg/mL (Figure 2). In another study, MBP (DBP metabolite) at the level of 10^−3^ M caused an increase of ROS level and impaired the developmental competency of preimplantation embryos [63]. In this study, a statistically significant increase in the hydroxyl radical level in RBCs treated with the parent phthalates at a concentration of 2.5 µg/mL was observed. However, in the case of their metabolites, such changes were observed at a higher concentration, corresponding to 10 µg/mL (Figure 3). Some researchers have suggested that, when present in adequate amounts, ROS act as signaling molecules by participating in signal transduction pathways and can induce changes in enzyme activity, apoptosis, and necrosis. Finally, this leads to pathological changes and organ dysfunction [35,64,65].

The main antioxidant enzyme that defends cells against ROS, including hydroxyl radical precursors, is SOD [56,66,67,68]. Our study showed that incubation of the erythrocytes with the selected phthalates caused a statistically significant decrease in SOD activity (inhibition of SOD activity increased along with increasing phthalates concentrations) (Table 2). Molecular docking studies conducted by Prasanth et al. also demonstrated that DBP and MBP had a concentration-dependent inhibitory effect on SOD [69]. The authors showed that DBP and MBP could bind the active site of SOD and create hydrogen bonds with the active site residue R143. This residue is crucial for the binding of ROS during their conversion to hydrogen peroxide and molecular oxygen. This may perhaps explain the inhibitory effect of DBP and MBP on SOD. The fact that MBP inhibited SOD activity at a concentration several times higher than the DBP concentration (Table 2) required to inhibit the same amount of enzyme, may be attributed to the smaller dimensions of the MBP molecule compared to DBP. Hence, MBP may occupy different positions with comparable binding energies, and in some of them, the ligand may not interact with R143 [69].

SOD-mediated reaction leads to the formation of H_2_O_2_, that is subsequently decomposed by CAT or GSH-Px to water and oxygen [56,68,70]. Therefore, we determined the activity of CAT and GSH-Px. Our analysis showed that by reducing the phthalates concentration, there was a slight decrease in CAT activity. However, the decrease was not statistically significant. Higher concentrations of phthalates caused a statistically significant increase in the activity of this enzyme (Table 2). The same variations in CAT activity were observed in earthworms after treatment with different doses of DBP and BBP. In in vivo systems, the increase in CAT activity is due to the increased expression of the enzyme under the influence of xenobiotics [36,71,72]. In contrast, in human erythrocytes, which do not possess a nucleus, the increase in CAT activity under the influence of the analyzed phthalates could be caused by oxidation of hemoglobin to metHb, because metHb shows catalase-like activity [73]. In our study, a statistically significant increase in CAT activity was observed at phthalates concentrations that caused a statistically significant increase in metHb level (Table 1 and Table 2). González-Sánchez et al. suggested that the catalase-like activity of methemoglobin must predominantly be a biocatalytic reaction that protects the protein against H_2_O_2_-induced suicide inactivation [74].

By releasing reduced form of nicotinamide adenine dinucleotide phosphate (NADPH), catalase in erythrocytes affects the system of glutathione-dependent enzymes (glutathione peroxidase/glutathione reductase), regulating their activity [75]. Under conditions of excessive ROS formation and metHb levels in erythrocytes, there may be a significant decrease in the level of NADPH, which plays a crucial role for the proper function of GSH-Px [76]. In this study, DBP and BBP caused a statistically significant decrease in GSH-Px activity at a concentration five times lower than that of their metabolites. We also observed that a reduction in the activity of this enzyme increased along with increasing concentrations of the analyzed phthalates (Table 2). A similar correlation was observed by Zhou et al. in an in vivo study on the epididymis of adult rats. GSH-Px is susceptible to inactivation by its own substrates (hydrogen peroxide, organic peroxides) [77]. The enzymatic performance of GSH-Px in RBCs drops (or comes to a complete halt) with exposure to high concentrations of H_2_O_2_. The reason for this is the slow regeneration rate of this enzyme by GSH reductase and thioredoxin [78,79,80].

Our study showed that phthalates such as DBP and BBP and their metabolites MBP and MBzP caused an increase in free radical production, which most likely led to the oxidation of Hb and a decrease in the activity of antioxidant enzymes. DBP and BBP caused statistically significant changes at lower concentrations (from 1 µg/mL) than their metabolites (5 µg/mL). This may be associated with the high lipophilicity of DBP and BBP and a high bio-accumulation potential, that we observe for chemical compounds with a value of log *P* > 3 (log *P* for DBP, BBP, MBP, and MBzP was 4.83, 5.00, 2.72, and 2.90, respectively) [81].

In summary, this study indicated that DBP, BBP, and their metabolites (MBP and MBzP) disturbed the redox balance in erythrocytes at concentrations that have been found in the human blood [13,14]. In addition, it is probable that oxidative stress induced by the studied phthalates in RBCs may contribute to accelerated eryptosis [37] and thus to the removal of the erythrocytes from the circulation. This, in turn, can lead to health complications in the form of anemia [82].

## 4. Materials and Methods

### 4.1. Chemicals

Phthalates: di-n-butyl phthalate (DBP), butylbenzyl phthalate (BBP); phthalate metabolites: mono-n-butylphthalate (MBP), monobenzylphthalate (MBzP) (99–99.5% purity) were purchased from Sigma-Aldrich (USA).

### 4.2. Isolation and Treatment of Human Erythrocytes

Erythrocytes were isolated from the leucocyte buffy coat separated from blood bought in the Regional Centre of Blood Donation and Blood Treatment in Łódź, Poland. Blood was taken from 25 anonymous healthy volunteers (aged 20–45). All procedures related to blood donation were executed at the Regional Centre of Blood Donation and Blood Treatment in Łódź, Poland. The blood donors recruitment was at the Centre, according to national legal procedures and European Union regulations (incl. the regulation (EU) 2016/679 OF THE EUROPEAN PARLIAMENT AND OF THE COUNCIL of 27 April 2016 on the protection of natural persons with regard to the processing of personal data and on the free movement of such data). The research studies were approved by the Bioethics Committee of the University of Lodz No. 16/KBBN-UŁ/III/2014. As agreed by the Bioethics Committee of the University of Lodz, no informed consent is needed for studies on bought anonymous human blood samples. All methods were performed in accordance with the relevant guidelines and regulations.

Leukocyte buffy coat was diluted using Ringer buffer and centrifuged at 3000 rpm for 10 min at 4 °C. The pellet was washed two times using Ringer buffer (5 mM KCl, 125 mM NaCl, 1 mM CaCl_2_, 1 mM MgCl_2_, 32 mM HEPES, 25 mM Tris 10 mM glucose, pH 7.4). RBC of 5% hematocrit were suspended in Ringer buffer, treated with DBP, BBP, and their metabolites (0.5–100 µg/mL) and incubated at 37 °C for 6 h or 24 h in total darkness. Control samples consisted of RBCs, which were incubated with Ringer buffer and ethanol (final concentration of ethanol, 0.2%) [37].

### 4.3. Hemoglobin Oxidation

After incubation, the samples were treated with deionized water and centrifuged (2400 rpm, 10 min, 4 °C). Absorbance of oxidized hemoglobin (MetHb) was measured at two wavelengths, i.e., 630 nm and 700 nm. Then, a solution containing potassium ferricyanide (1 M Fe 2+: 3 M K_4_[Fe(CN)_6_]) was added to the hemolysate containing methemoglobin, and the samples were re-assayed for absorbance at the same wavelengths (positive control) [83].

The percent of methemoglobin was calculated by the following Equation (1):

(1) $$\mathrm{MetHb}\;\left[\%\right]=\frac{\left(A_{630}-\;A_{700}\right)}{(A_{100\%\mathrm{metHb}\;630}-\;A_{100\%\mathrm{metHb}\;700})}\times 100\%$$

where:

MetHb [%], percentage of hemoglobin

A_630_, absorbance of Hb in the sample tested at 630 nm

A_700_, absorbance of Hb in the sample tested at 700 nm

A_100%metHb 630_, absorbance of Hb at 630 nm after treatment with potassium ferricyanide

A_100%metHb 700_, absorbance of Hb at 700 nm after treatment with potassium ferricyanide

### 4.4. Oxidation of H_2_DCFDA and HPF

After 6 h of incubation with DBP, BBP, MBP, or MBzP, the cells were centrifuged (600 *g* for 10 min at 4 °C) and diluted with PBS (final density 1 × 10^6^ cells/mL). Then, the cells were incubated with the fluorescent probe 6-carboxy 2′,7′-dichlorodihydrofluorescein diacetate (H_2_DCFDA) (at a final concentration of 5 μM) or 3′-(p-hydroxyphenyl)-fluorescein (HPF) (at a final concentration of 4 μM) for 20 min at 37 °C in total darkness. In order to measure the production of ROS, the increase in fluorescence of dichlorofluorescein (DCF) (a marker of H_2_DCFDA oxidation) was measured by a flow cytometer (LSR II. Becton Dickinson) at excitation/emission wavelengths of 488 nm and 530 nm, respectively. The increase in fluorescence of HPF (highly reactive oxygen species detection) was measured at excitation/emission wavelengths of 490 nm and 515 nm, respectively. The analysis of 10,000 cells was performed [84].

### 4.5. Superoxide Dismutase Activity

The analysis of SOD (EC 1.15.1.1) activity is based on the ability of SOD to inhibit the process of epinephrine self-oxidation in an alkaline medium according to the method of Misra and Firidovich [85]. In the reaction of colored adrenochrome formation, the superoxide anion radical is formed as an intermediate product. SOD activity was measured by monitoring the increase of absorbance at 480 nm and calculated in U/g hemoglobin (Hb) [83].

### 4.6. Catalase Activity

CAT activity analysis is based on the direct measurement of the rate of decomposition of hydrogen peroxide by catalase and hemoglobin present in a hemolysate, at a wavelength of 240 nm. The amount of enzyme that decomposes 1 µmol of hydrogen peroxide during 1 min corresponds to a unit of CAT activity. CAT activity was calculated in µmol/min/mg Hb [83].

### 4.7. Glutathione Peroxidase Activity

GSH-Px (E.C.1.11.1.9) activity was analyzed using tert-butyl peroxide as a substrate according to the method of Rice-Evans et al. [86]. Potassium azide was added to inhibit catalase activity, whereas potassium ferricyanide was added in order to inhibit the pseudoperoxidase activity of hemoglobin. The conversion of NADPH to NADP+ was determined at 340 nm at 25 °C for 3 min and calculated in µmol/min/g Hb [83].

### 4.8. Statistical Analysis

The results are shown as mean ± SD, achieved from 5 individual experiments (5 blood donors). Multiple comparisons among the group mean differences were analyzed by one-way analysis of variance (ANOVA) followed by Tukey’s post-hoc test. When the *p* value was lower than 0.05, the differences were considered to be statistically significant (*). The “sample size” and the “power of test” for all the data were checked. Statistical analysis was conducted using STATISTICA software ver.13 (StatSoft Inc., Tulsa, OK USA) [39].

## 5. Conclusions

1. Our study for the first time illustrates the mechanism of the oxidative action of DBP and BBP and their metabolites in non-nucleated human mature erythrocytes.

2. The compounds studied increased ROS (including ^•^OH) levels and altered the activities of the antioxidative enzymes SOD, CAT, and GSH-Px.

3. A comparison of the actions of phthalates and their metabolites showed that the parent compounds exhibited a stronger oxidative potential in red blood cells.

4. Changes in the parameters studied occurred at phthalates concentrations that may affect the human organism during environmental exposure [13,14].

5. The studied phthalates disturbed the redox balance in human erythrocytes, which may affect eryptosis [37] and thus results in accelerated removal of these cells from the circulation.

## Figures and Tables

**Table 1:** Changes in methemoglobin level in control erythrocytes and in erythrocytes incubated for 24 h with DBP, BBP, MBP, and MBzP used at concentrations ranging from 0.5 to 100 μg/mL.

Concentration	Methemoglobin (%)			
(µg/mL)	DBP	BBP	MBP	MBzP
0	1.21 ± 0.65	1.21 ± 0.65	1.21 ± 0.65	1.21 ± 0.65
0.5	3.19 ± 1.12	2.71 ± 0.76	2.11 ± 0.46	2.87 ± 0.73
1	3.10 ± 0.85	3.66 ± 0.77	2.45 ± 0.73	3.66 ± 0.43
2.5	6.61 ± 0.83 *	3.73 ± 0.44	2.70 ± 0.96	3.32 ± 0.43
5	8.99 ± 0.94 *	4.83 ± 0.81 *	2.86 ± 0.97	4.08 ± 0.48
10	16.85 ± 1.67 *	13.99 ± 1.10 *	3.66 ± 0.81	4.70 ± 0.97
20	25.15 ± 1.87 *	24.94 ± 2.79 *	5.09 ± 0.65	5.68 ± 0.91
50	-	-	7.73 ± 0.80 *	9.74 ± 1.09 *
100	-	-	9.90 ± 0.89 *	14.09 ± 2.23 *

Legend: DBP, di-n-butyl phthalate; BBP, butylbenzyl phthalate; MBP, mono-n-butylphthalate; MBzP, monobenzylphthalate; “-” no data; (*) *p* < 0.05.

**Table 2:** Changes in the activity of SOD, CAT, and GSH-Px in control erythrocytes and in erythrocytes incubated for 24 h with DBP, BBP, MBP, and MBzP used at concentrations ranging from 0.5 to 100 μg/mL.

Compound	Concentration (µg/mL)	Activity of Antioxidant Enzymes		
		SOD (U/g Hb)	CAT (µmol/min/mg Hb)	GSH-Px (µmol/min/gHb)
	0	1373.9 ± 139.2	161.97 ± 5.6	32.0 ± 3.6
DBP	0.5	1220.1 ± 79.5	159.7 ± 11.8	31.7 ± 2.5
	1	1145.1 ± 68.6	168.7 ± 21.5	27.3 ± 3.1
	2.5	1028.1 ± 79.6 *	181.3 ± 16.0	23.5 ± 2.2
	5	920.5 ± 56.3 *	215.9 ± 12.0 *	21.6 ± 3.2 *
	10	754.1 ± 44.6 *	220.7 ± 18.6 *	18.9 ± 5.4 *
	20	662.5 ± 82.2 *	238.8 ± 15.5 *	17.9 ± 6.2 *
BBP	0.5	1279.1 ± 89.7	151.5 ± 8.5	30.8 ± 4.9
	1	1165.1 ± 64.1	166.0 ± 14.3	30.4 ± 4.4
	2.5	904.6 ± 61.5 *	179.2 ± 12.3	28.5 ± 4.2
	5	841.9 ± 75.5 *	197.9 ± 13.2 *	22.7 ± 3.8*
	10	750.0 ± 95.3 *	207.1 ± 15.4 *	19.2 ± 5.1 *
	20	549.7 ± 66.6 *	231.5 ± 14.6 *	18.4 ± 5.6 *
MBP	2.5	1270.2 ± 78.5	154.1 ± 15.9	31.3 ± 5.7
	5	1177.7 ± 105.3	158.6 ± 17.1	26.1 ± 5.8
	10	837.1 ± 64.0 *	165.1 ±16.8	24.6 ± 5.5
	20	759.1 ± 82.9 *	184.6 ± 10.9	23.9 ± 1.7
	50	673.2 ± 89.4 *	201.3 ± 22.5 *	21.6 ± 1.9 *
	100	436.9 ± 59.7 *	217.2 ± 20.9 *	20.6 ± 1.9 *
MBzP	2.5	1225.7 ± 122.5	156.8 ± 19.4	33.1 ± 4.4
	5	1046.1 ± 149.7	160.4 ± 12.8	28.1 ± 6.3
	10	861.0 ± 95.2 *	179.8 ± 17.8	27.6 ± 2.8
	20	815.1 ± 87.3 *	185.4 ± 13.6	24.9 ± 3.1
	50	631.2 ± 98.1 *	216.6 ± 14.5 *	24.8 ± 6.1 *
	100	577.8 ± 67.5 *	222.0 ± 19.1 *	23.7 ± 3.6 *

Legend: SOD, superoxide dismutase; CAT, catalase; GSH-Px, glutathione peroxidase; (*) *p* < 0.05.

## Abbreviations

BBP	Butylbenzyl phthalate
BHb	Bovine hemoglobin
CAT	Catalase
DBP	Di-n-butyl phthalate
DCF	Dichlorofluorescein
ECHA	European Chemicals Agency
EDCs	Endocrine-Disrupting Chemicals
EU	European Union
GSH- Px	Glutathione peroxidase
H_2_DCFDA	6-carboxy 2′,7′-dichlorodihydrofluorescein diacetate
Hb	Hemoglobin
hHb	Human hemoglobin
HPF	3′-(p-hydroxyphenyl)-fluorescein
HTC	Hematocrit
K_ow_	Octanol–water partition coefficient
MBP	Mono-n-butylphthalate
MBzP	Monobenzylphthalate
metHb	Methemoglobin
NADPH	Reduced form of Nicotinamide Adenine Dinucleotide Phosphate
PAEs	Phthalates
PBMCs	Peripheral Blood Mononuclear Cells
RBCs	Red Blood Cells
ROS	Reactive Oxygen Species
SOD	Superoxide dismutase
SVHC	Substances of Very High Concern
^•^OH	Hydroxyl radical
